# Multidisciplinary Approach to Ventricular Arrhythmias in the CICU: Integrating Mechanical Circulatory Support, Ablation, and Emerging Therapies

**DOI:** 10.3390/jcm15093459

**Published:** 2026-05-01

**Authors:** Alfredo Mauriello, Adriana Correra, Anna Chiara Maratea, Valeria Cetoretta, Francesco Giallauria, Giovanni Esposito, Alfonso Desiderio, Francesco Sabatella, Gemma Marrazzo, Biagio Liccardo, Vincenzo Russo, Paolo Trambaiolo, Antonello D’Andrea

**Affiliations:** 1 S.C. Cardiology, Institute National Cancer, IRCCS, Fondazione “G. Pascale”, Via M. Semmola 52, 80131 Naples, Italy; alfredo.mauriello93@libero.it; 2 Cardiology Department, Ospedali Riuniti University Hospital, Viale Pinto 1, 71122 Foggia, Italy; adrianacorrera@gmail.com; 3 Department of Cardiovascular Disease, ASL Napoli 1 Centro, Via Comunale del Principe, 13/a, 80145 Naples, Italy; annachiara.maratea@gmail.com; 4 Cardiology and Arrhythmology Clinic, Marche Polytechnic University, University Hospital “Ospedali Riuniti”, Via Conca 71, 60126 Ancona, Italy; vcetoretta@gmail.com; 5 Department of Translational Medical Sciences, “Federico II” University of Naples, Via S. Pansini 5, 80131 Naples, Italy; francesco.giallauria@unina.it (F.G.); giovanni.esposito2@unina.it (G.E.); 6 Cardiology and Intensive Care Unit, Department of Cardiology, “Umberto I” Hospital, Via Alfonso De Nicola 1, 84014 Nocera Inferiore, Italy; alf.desiderio@tiscali.it (A.D.); frasab93@gmail.com (F.S.); gemmamarrazzo@gmail.com (G.M.); 7 Cardiology Unit, Department of Medical and Translational Sciences, University of Campania “Luigi Vanvitelli”, “V. Monaldi” Hospital, Via Leonardo Bianchi SNC, 80131 Naples, Italy; liccardob@gmail.com (B.L.); vincenzo.russo@unicampania.it (V.R.); 8 Intensive Cardiac Care Unit, Department of Cardiology, Sandro Pertini Hospital, Via Monti Tiburtini, 287, 00157 Rome, Italy; paolo.trambaiolo@gmail.com

**Keywords:** ventricular arrhythmias, electrical storm, cardiac intensive care units, ventricular fibrillation, ventricular tachycardia

## Abstract

**Background/Objectives**: The management of ventricular arrhythmias (VAs) within cardiac intensive care units (CICUs) is undergoing a significant transformation. This review aims to analyze the historical transition from a narrow focus on arrhythmia-specific treatments toward on the multidisciplinary heart rhythm team. **Methods**: A narrative revies was conducted. **Results**: Effective management of electrical storm (ES) requires prompt attenuation of sympathetic hyperactivity, with a preference for non-selective beta-blockers and the implementation of deep sedation. The use of mechanical circulatory support (MCS) has emerged as a mechanical antiarrhythmic strategy by facilitating ventricular unloading and reducing myocardial wall stress. Furthermore, early catheter ablation, guided by 3D electroanatomical mapping and advanced imaging, has proven superior to salvage procedures for stabilizing the arrhythmic substrate. Finally, the integration of palliative care ensures ethical stewardship during refractory shock. **Conclusions**: Modern VAs management in the CICUs represents a convergence of technology, biology, and multidisciplinary coordination.

## 1. Introduction

In recent years, we have witnessed significant changes in the cardiac patient population, partly due to aging, but primarily because of improvements in healthcare and their impact on the natural history of various cardiovascular diseases.

These changes have led to an increase in the number of patients requiring acute cardiac care, with progressively more complex cardiovascular pathologies, often complicated by acute or chronic non-cardiovascular comorbidities, which impact both the management and prognosis of these patients [1].

Consequently, the coronary care units of the past, which played a fundamental role in treating and improving the prognosis of patients with acute myocardial infarction (MI), the contemporary management of the critical cardiac patient requires highly specialized healthcare provided in dedicated cardiac intensive care units (CICUs) [2,3].

Within the intensive care setting, cardiac arrhythmias represent a ubiquitous clinical challenge, manifesting either as the precipitating cause for admission or as a complication of systemic critical illness [4]. While supraventricular disturbances remain more prevalent, ventricular arrhythmias (VAs), accounting for approximately 2% of primary admissions, affect nearly 8.6% of the broader ICU population during their clinical course [4,5]. The clinical significance of these malignant rhythms cannot be overstated, as recurrent ventricular episodes serve as a primary catalyst for hemodynamic collapse, irreversible anoxic encephalopathy, and increased rates of in-hospital mortality [6]. Compared to patients with no arrhythmia, patients experiencing unclustered VAs had a 2.7-fold increased risk of death [7]. Therefore, this narrative review aims to explore the historical transition from a narrow focus on arrhythmia-specific treatment toward a more holistic, integrated management strategy.

## 2. Materials and Methods

This narrative review provides a comprehensive synthesis and critical analysis of the current knowledge, clinical advancements, and future perspectives concerning the management of VA within the CICUs. Narrative reviews are particularly effective for synthesizing broad and evolving research domains, such as the historical transition from isolated arrhythmia treatments to integrated, holistic management strategies.

To identify relevant sources, a structured literature search was conducted focusing on the key conceptual themes of the review and related clinical challenges. The search strategy utilized the biomedical databases PubMed/MEDLINE (Bethesda, Maryland, MD, USA) and EMBASE (Amsterdam, The Netherlands) for publications ranging from January 2015 to January 2026, adhering to standard practices for evidence-based reviews in cardiovascular medicine.

The search strings and key terms employed for literature collection included combinations of the following concepts, utilizing Boolean operators (AND/OR):

Primary Condition: “Ventricular arrhythmias” (VAs), “Electrical storm” (ES), “Ventricular tachycardia” (VT), “Ventricular fibrillation” (VF).

Clinical Setting: “Cardiac Intensive Care Units” (CICUs), “Critical cardiac patient”, “Hemodynamic collapse”.

Therapeutic Strategies: “Anti-arrhythmic drugs”, “Deep sedation”, “Mechanical circulatory support” (MCS), “Catheter ablation”, “Neuromodulation”.

Document selection prioritized English-language articles, including systematic and narrative reviews, retrospective and prospective studies, and randomized controlled trials (RCTs) that provided insights into the pathophysiology, clinical evidence, and technological innovations in managing acute arrhythmic crises. While this process does not strictly adhere to the rigorous replicability of a formal systematic review, this methodology ensures that the narrative synthesis is evidence-based and encompasses the major clinical and scientific developments related to the multidisciplinary heart rhythm team.

## 3. Acute Management: Beyond Defibrillation

### 3.1. Acute Stabilization Protocols: Pharmacological Strategies

#### 3.1.1. Structural Heart Disease

Anti-arrhythmic drugs are the cornerstone of therapy for the acute management of ES. The selection of an optimal antiarrhythmic drug regimen is a nuanced process, dictated by the specific morphology of the ventricular arrhythmia and its presumed underlying pathophysiology. This therapeutic strategy must be meticulously tailored to the individual’s clinical profile, accounting for systemic comorbidities, potential pharmacological contraindications, and the immediate availability of specific agents within the acute setting.

In the setting of structural heart disease, the attenuation of sympathetic hyperactivity serves as a cornerstone of therapy [8]. First-line management necessitates the prompt administration of beta-blockers (BB), with a distinct preference for non-selective agents such as propranolol, which offer superior suppression of the peripheral adrenergic surge, provided no absolute contraindications are present [9].

In a randomized controlled trial (RCT) [10], including 60 patients with implantable cardioverter defibrillator (ICD) patients (45 men, mean age 65.0 ± 8.5 years), the combination of i.v. amiodarone and oral propranolol were safe, effective, and superior to the combination of i.v. amiodarone and oral metoprolol in the management of ES in ICD patients (incidence rate ratio of ventricular arrhythmias: 0.375; 95% confidence interval (CI): 0.207 to 0.678; *p* = 0.001).

In 49 patients (73% men, mean age 57 ± 10 years), including in RCT, with an ES after a recent (<3 months) MI, sympathetic blockade with i.v. esmolol or i.v. propranolol (160–320 mg/24 h) or left stellate ganglion blockade was superior to lidocaine i.v for VAs suppression and survival (*p* < 0.0001) [9].

In a retrospective study, including 20 patients (85% male; aged 69.8  ±  9.8 years) with recurrent ventricular arrhythmias in the early post-MI, quinidine has been reported to be effective after BB and amiodarone treatment failure. During long-term follow-up (range 2 months to 11 years), patients not receiving quinidine developed recurrent polymorphic VT, while there were no recurrent arrhythmias during long-term quinidine therapy. Therefore, in the early post-MI period, quinidine represents an excellent alternative when beta-blockers and amiodarone fail.

In patients with recurrent, haemodynamically unstable ventricular arrhythmias despite amiodarone, landiolol, an ultra-short-acting β1-selective blocker, was effective for arrhythmia suppression initially in two smaller studies [11,12]. Therefore, in post-marketing surveillance [13], landiolol has shown safety and effectiveness in real-world settings in Japan, including 253 patients.

In the management of ES within the context of structural heart disease, intravenous amiodarone remains a cornerstone intervention. Clinical evidence suggests that a bolus of 5 mg/kg over 20 min effectively terminates hemodynamically tolerated ventricular tachycardia in approximately 38% of cases, primarily in those with underlying structural heart disease [14]. Notably, the integration of concomitant intravenous and oral loading may accelerate rhythm stabilization and reduce the total cumulative dose compared to monodal oral administration [15]. Furthermore, for patients already maintained on chronic amiodarone, acute reloading serves as a viable and often effective strategy to regain electrical control during breakthrough crises [16].

In cases of hemodynamically tolerated ventricular tachycardia without acute ischemia, procainamide emerges as a robust alternative. Data from the PROCAMIO trial [14], including 62 patients (mean 64 ± 16 years), highlight its superiority over amiodarone, demonstrating higher termination rates for VT of unknown origin alongside a more favorable safety profile (OR: 0.17; 95% CI: 0.04–0.73, *p* = 0.017). Conversely, lidocaine exhibits only modest efficacy in stable monomorphic ventricular tachycardias, trailing behind both procainamide [15] and sotalol [16]; however, it retains a specific niche in ischemia-driven arrhythmias due to its minimal negative inotropic impact [17]. Emerging evidence also supports the use of ranolazine [18]; beyond its antianginal properties, it exerts antiarrhythmic effects by inhibiting late sodium (NaL) and delayed rectifier potassium (Kr) currents. In the Ranolazine in High-Risk Patients With Implanted Cardioverter-Defibrillators (RAID) trial, including 1012 ICD patients (82% male; mean age was 64 ± 10 years), ranolazine significantly attenuated the burden of recurrent ventricular arrhythmias in ICD recipients with structural heart disease, without adversely affecting mortality (95% CI: 0.67 to 1.05; *p* = 0.117).

#### 3.1.2. Primary Electrical Disease

In patients with primary electrical diseases, the management of ES is strictly phenotype specific.

For Long QT Syndrome (LQTS), immediate intervention focuses on the elimination of potential triggers and the administration of non-selective beta-blockers, such as nadolol or propranolol. In both acquired and congenital forms, aggressive supplementation of extracellular magnesium and potassium is mandatory [19,20]. However, further management diverges: acquired LQTS (aLQTS) may require isoproterenol or overdrive pacing to suppress pause-dependent triggers, whereas congenital LQTS (cLQTS) Type 3 often necessitates mexiletine [21]. Crucially, Class III antiarrhythmic drugs must be avoided to prevent further QT prolongation [21].

In Brugada Syndrome (BrS) [22] and idiopathic ventricular fibrillation (IVF) [23], isoproterenol remains the therapeutic gold standard for suppressing ES, occasionally supplemented by quinidine.

Conversely, catecholaminergic polymorphic ventricular tachycardia (CPVT) represents a unique challenge where the adrenergic surge is the primary driver; here, therapy centres on the radical withdrawal of catecholamines, including the avoidance of epinephrine even during resuscitation and the absolute reliance on high-dose beta-blockade [24].

In Table 1 are summarized the antiarrhythmic drugs, classified sec. Vaughan William Classification, with an explanation of the pharmacokinetic and pharmacodynamic profiles, and therapeutic indication.

### 3.2. The Significance of Autonomic Equilibrium: Impact of Deep Sedation and Neuromuscular Blockade

The cardiac autonomic nervous system, in particular the sympathetic nervous system, is recognized to play a critical pathophysiologic role in VAs, and neural modulation through various avenues is increasingly gaining attention; however, the detailed electrophysiological mechanisms mediated through the cardiac autonomic nervous system are not completely understood [26].

In conscious patients burdened by recurrent ICD shocks or symptomatic VAs, the implementation of mild-to-moderate sedation is a critical adjunct for both symptomatic relief and the attenuation of the pro-arrhythmic adrenergic surge. For cases of drug-refractory ES, escalating to deep sedation and mechanical ventilation has proven to be a highly effective rescue strategy [27,28]. Data from a diverse cohort of 116 patients (65.6 years, 53.5–74.3) with refractory ES indicate that deep sedation achieved acute rhythm stabilization defined as immediate cessation with 24 h suppression, in 47% of cases. Crucially, the achievement of an acute clinical response following deep sedation has emerged as an independent predictor of improved in-hospital survival, underscoring the prognostic value of quieting the autonomic nervous system [27]. Table 2 summarizes the drugs used in CICUs for sedation regarding the management of VAs.

Neuromodulation of cardiac autonomic nervous system for the termination of ventricular arrhythmia storm has been promising, including surgical cardiac sympathetic denervation [29], stellate ganglion blockade [30], renal sympathetic denervation [31] and thoracic epidural anaesthesia [32].

In a retrospective study [29], including 41 patients (85% male; mean age 59 ± 13 years) who underwent left and bilateral cardiac sympathetic denervation, there was a significant reduction in the burden of ICD shocks during follow-up compared to the 12 months before the procedure (*p* < 0.001). Therefore, shock-free survival was greater in the bilateral group than in the left cardiac sympathetic denervation group (*p* = 0.04)

In the STAR study, a multicentric RCT, including 131 patients (83.2% male; mean age 68, 63.8–69.2 years), the author showed that arrhythmic episodes requiring treatment were significantly reduced comparing 12 h before the first percutaneous stellate ganglion block with 12 h after the last procedure [*p* < 0.0001] and comparing 1 h before with 1 h after each procedure [*p* < 0.001]. Percutaneous stellate ganglion block is a rapid and underutilized intervention that can immediately stabilize the patient in the CICU before proceeding to more invasive solutions.

Bazoukis et al. [31], in their systematic review including 34 animal and human studies, showed that renal denervation can modulate ventricular electrophysiological properties and exerts favorable effects in the development and recurrence of VAs.

Do et al. [32], in their small case series, including 11 patients, showed that more than half of the patients with VAs responded to thoracic epidural anesthesia.

## 4. Mechanical Circulatory Support and Arrhythmias

### 4.1. Technological Advances: Utilizing Impella, IABP, and VA-ECMO for Hemodynamic Support in the Context of Incessant Arrhythmias

Current temporary modalities for MCS include the intra-aortic balloon pump (IABP), the Impella devices (Abiomed, Danvers, MA, USA), Tandem Heart left atrial-to-femoral bypass (Cardiac Assist Inc., Pittsburgh, PA, USA), extracorporeal membrane oxygenation (ECMO), and peripheral cardiopulmonary bypass. Their main characteristics are depicted in Table 3.

Owing to its rapid deployment and comprehensive circulatory support, veno-arterial extracorporeal membrane oxygenation (VA-ECMO) remains the most compelling strategy for patients in hemodynamically unstable ventricular arrhythmia [35]. While initial RCTs demonstrated a dramatic reduction in mortality compared to conventional cardiopulmonary resuscitation (7% vs. 43%) [36], subsequent larger multicenter trials have tempered this enthusiasm [37,38].

Suverein et al. [38], in their RCT, including 160 patients (75% male; mean age 56 ± 12 years), showed that extracorporeal CPR was not superior to conventional CPR in terms of mortality at 30 days (20 vs. 16%, *p* = 0.52). At the same, Belohlavek et al. [37], in their RCT, including 256 patients (83% male; median age was 59 years, 48–66) with out-of-hospital cardiac arrest (OHCA) of presumably cardiac cause, showed that extracorporeal CPR failed to show a significant benefit in mortality when compared with conventional CPR (32 vs. 22%, *p* = 0.09).

These conflicting outcomes underscore that the success of an extracorporeal CPR program is not merely a matter of technology, but rather the systematic selection of optimal candidates and the refinement of peri-procedural care. Meanwhile, the role of alternative MCS platforms, such as the Impella pump, remains poorly defined and limited to anecdotal evidence from small case series.

### 4.2. Ventricular Unloading: Reducing Wall Stress Through MCS as a Mechanical Antiarrhythmic Strategy

The implementation of MCS in the setting of ES transcends mere hemodynamic stabilization; it functions as a potent mechanical antiarrhythmic strategy. By facilitating ventricular unloading, MCS devices effectively reduce myocardial wall stress. This reduction in diastolic tension blunts the activation of stretch-activated ion channels, which are known to trigger delayed afterdepolarizations (DADs) and exacerbate localized reentry [39]. Consequently, the mitigation of regional myocardial stretch elevates the ventricular fibrillation threshold and suppresses the triggers of incessant ventricular tachycardia [39]. This mechanical-electrical decoupling is particularly vital during complex ablations, where maintaining a quiescent and unloaded ventricle can prevent the initiation of hemodynamically catastrophic rhythms.

### 4.3. Bridge to Treatment: Utilizing Mechanical Support for High-Risk Catheter Ablation

Acute hemodynamic deterioration occurs in approximately 11% of patients undergoing catheter ablation for ventricular tachycardia, serving as a potent marker for increased both short- and long-term mortality. While VA-ECMO is the primary rescue modality for MCS during ES, outcomes remain sobering; contemporary series report mortality rates between 38% and 76%, even when the ablation procedure itself is technically successful [40,41].

In this context, the PAINESD score (Table 4) serves as a vital prognostic tool, enabling clinicians to estimate the risk of peri-procedural hemodynamic collapse in scar-related ventricular arrhythmia cases [42]. The likelihood of decompensation is further modulated by the procedural workflow, particularly when extensive activation mapping or repetitive ventricular arrhythmia induction is anticipated. Current data, including a meta-analysis of 19 non-randomized studies over 2400 patients with ES or high PAINESD scores (> 15), suggest that prophylactic MCS may offer a significant survival advantage (*p* < 0.01) [43]. However, this benefit must be weighed against a heightened risk of vascular complications and increased procedural complexity, including prolonged fluoroscopy times [44]. Consequently, a comprehensive pre-procedural strategy, addressing vascular access, the necessity for left ventricular venting, and the projected duration of support, is essential to optimize outcomes.

## 5. Emerging Frontiers: Early Catheter Ablation for the Management of Acute Arrhythmic Crises

### 5.1. Timing of Intervention: Shifting from Salvage Ablation to Early Procedures to Prevent Electrical Remodeling

In patients with structural heart disease presenting with ES and monomorphic VT in the absence of reversible triggers, catheter ablation during the index hospitalization is strongly indicated [45]. Early intervention serves to acutely stabilize incessant arrhythmia. However, successful outcomes are contingent upon three critical pre-procedural pillars. First, hemodynamic optimization is paramount; since mapping often requires tachycardia induction under sedation, pre-emptive stabilization with vasopressors or mechanical circulatory support (MCS) is essential to prevent intraprocedural decompensation [43]. Second, while trigger suppression remains a priority, ablation is still necessary to neutralize the underlying arrhythmogenic substrate [45]. Finally, the nature of the substrate profoundly influences mapping strategy; while ischemic cardiomyopathy typically presents more localized circuits, non-ischemic cardiomyopathy often requires epicardial access [46]. Despite the inherent complexity and higher recurrence rates in specific phenotypes like sarcoidosis, ablation remains a lifesaving maneuver across diverse etiologies, including Brugada syndrome and idiopathic ventricular fibrillation triggers [47].

### 5.2. Advanced Mapping in the CICUs: Integration of 3D Electroanatomical Mapping in High-Acuity Scenarios

The integration of 3D electroanatomical mapping systems, such as Carto 3 (Abbott, St. Paul, MN, USA), Ensite Precision (Abbott, St. Paul, MN, USA), and Rhythmia (Boston Scientific, Marlborough, MA, USA), into critical care scenarios has revolutionized the management of complex VA, enabling high-fidelity anatomical and electrical reconstruction of the ventricles [48]. In high-acuity clinical settings, the deployment of multipolar mapping catheters, such as Pentaray (Biosense Webster Inc., Diamond Bar, CA, USA), HD Grid (Abbott, St. Paul, MN, USA), and Orion (Boston Scientific, Marlborough, MA, USA), allows for the rapid acquisition of thousands of activation points, thereby enhancing the identification of the arrhythmic substrate while significantly reducing fluoroscopic exposure [48].

A significant challenge in these scenarios is the anatomical cardiac displacement induced by premature ventricular contractions. Such displacement may lead to operator error regarding the true localization of the arrhythmia’s site of origin. To circumvent this, the integration of advanced modules like LAT Hybrid enables the automatic positional correction of mapped points during a PVC based on the coordinates of the preceding sinus beat, consequently improving the mid-term efficacy of ablative lesions [49].

Furthermore, in patients with implanted devices, the integration of single photon emission computed tomography (SPECT)/computed tomography (CT) imaging into mapping systems allows for the precise delineation of ischemic scar zones without the artifacts typically associated with cardiac magnetic resonance image (MRI) [50], facilitating targeted ablation planning. Finally, the concurrent use of intracardiac echocardiography (ICE) provides real-time visualization of catheter stability and tissue contact points, optimizing procedural safety in high-risk patients [50,51].

### 5.3. Image-Guided Ablation: Utilizing Cardiac MRI and CT for Pre-Procedural Identification of the Arrhythmic Substrate

The clinical application of MRI and CT has become fundamental for the pre-procedural characterization of the arrhythmic substrate, enabling the precise identification of scar extent and heterogeneous border zones. Late gadolinium enhancement MRI (LGE-MRI) is regarded as the gold-standard technique for defining 3D scar architecture and distinguishing between the dense infarct core and the heterogeneous tissue that facilitates re-entry, thereby enhancing arrhythmic risk stratification [52].

Through image integration, structural data derived from MRI are registered onto EAM to guide ablation toward critical conduction channels. This strategy reduces the total number of radiofrequency applications and improves long-term procedural efficacy [52]. In patients with implantable devices that generate significant artefacts, or where MRI is contraindicated, cardiac CT serves as a robust alternative to analyze myocardial wall thinning and localize the cicatricial substrate [53].

These technologies not only streamline procedural planning, allowing for an informed decision between endocardial or epicardial access, but also minimize the requirement for activation mapping during unstable arrhythmias, significantly enhancing both the safety and precision of the intervention [54]. While advanced substrate characterization via LGE-MRI or CT offers an unparalleled anatomical roadmap for ablation, the logistical challenge of transporting a hemodynamically unstable patient in the throes of an ES often renders pre-procedural cross-sectional imaging unattainable. In such high-acuity scenarios, the integration of ICE becomes indispensable, providing real-time, bedside visualization of myocardial thickness, catheter contact, and potential complications, effectively bridging the diagnostic gap without compromising patient safety through hazardous intra-hospital transport.

## 6. Emerging Frontiers: Non-Invasive Therapeutic Strategies

### 6.1. Stereotactic Arrhythmia Radioablation (STAR): The Use of Radiation for the Non-Invasive Treatment of Refractory Ventricular Tachycardia

Stereotactic arrhythmia radiotherapy (STAR) is suggested as potentially effective and safe treatment for patients with therapy-refractory ventricular tachycardia. van der Ree et al. [55], in STARNL-1 trial, including six male patients with an ischaemic cardiomyopathy, were enrolled, and median age was 73 years (range 54–83), showed that a measure of ≥50% reduction in treated ventricular tachycardia-episodes at the end of 12-month follow-up was achieved in four patients (67%).

At the same time, Siklody et al. [56], in their retrospective study, including 20 patients (75% male; mean age 68, 47–80 years), showed that during the first 6 months after STAR, ventricular tachycardia burden decreased by 92%.

### 6.2. Genetics and Pharmacogenomics: Advancing Precision Medicine for Acute Arrhythmic Crises

Beyond specific syndromes, such as congenital long QT syndromes, or BrS, some different polymorphisms can have a role in drug response [57]. Pharmacogenetics plays a crucial role in understanding the variability in response to drug therapy, which is often attributed to genetic differences in drug metabolism and disposition. A prominent example with high relevance to the management of cardiac arrhythmias is some alleles of the CYP2D6 gene, which encodes a hepatic cytochrome P450 enzyme responsible for the metabolism of approximately 25% of clinically used drugs, including the beta-blockers metoprolol, as well as antiarrhythmic drugs such as propafenone [58]. In addition, rapidly identifying a mutation in the ryanodine receptor (RyR2) in patients with CPVT is a life-saving diagnostic step. This genetic insight allows clinicians to strictly avoid the administration of epinephrine during resuscitation, which would be counterproductive and potentially lethal in this population and instead prompts the immediate initiation of targeted therapies such as nadolol or flecainide [59]. Regarding side effects, a study showed that the risk of amiodarone-induced thyrotoxicosis type 2 is 3.18 times higher in the G/T of the DUOX1 gene carriers [60].

### 6.3. Inflammation and Fibrosis: Anti-Inflammatory and Anti-Fibrotic Strategies

Myocardial inflammation is often associated with ventricular arrhythmias [61]. The mechanisms underlying the arrhythmogenesis in myocardial inflammation are currently not fully understood [62]. A key role appears to be played by the nucleotide-binding oligomerization domain-like receptor family pyrin domain containing 3 (NLRP3) inflammasomes [63]. Once activated by stress, ischemia, or metabolic disease, it releases interleukin (IL)-1β, tumor necrosis factor (TNF)-α and IL-18, which not only promote more fibrosis but also cause immediate electrical instability, directly disrupt calcium handling (Ca^2+^) and potassium currents (K^+^). This shortens the action potential duration and promotes early afterdepolarizations (EADs) [63]. In addition, inflammation leads to an increase in oxidative stress. The ryanodine receptor 2 (RyR2), the cardiac muscle-specific isoform responsible for Ca^2+^-mediated Ca^2+^ release from sarcoplasmic reticulum, is also prone to oxidative stress. A number of preclinical studies have shown that reactive species of oxygen (ROS) promote RyR sulfhydryl oxidation leading to reduced Ca^2+^ transients and enhanced sarcoplasmic reticulum Ca^2+^ leak and thereby increase the risk for VAs [64,65,66,67].

Myocardial fibrosis—whether of ischemic [68] or non-ischemic origin—is widely established to be associated with the onset of ventricular arrhythmias.

In this context, several molecules, currently undergoing preclinical and clinical studies, have been proposed to reduce the inflammatory and fibrotic burden. Some molecules act both directly on NLRP3 [69] and indirectly, by blocking downstream interleukins—such as Anakinra or Canakinumab (anti-IL1β)—or colchicine [70]. Meanwhile, Pirfenidone [71] and sodium glucose cotransporter 2 inhibitors have been proposed as agents to reduce intramyocardial fibrosis.

## 7. A Multidisciplinary Approach: The Role of the Heart Rhythm Team

### 7.1. Synergy Among Intensivists, Electrophysiologists, and Cardiac Surgeons in Arrhythmia Management

The management of refractory ES has undergone a fundamental shift, moving away from isolated bedside attempts toward a centralized, multidisciplinary Heart Rhythm Team model. This collaborative framework is predicated on the understanding that ES is a systemic crisis, where electrophysiological instability, hemodynamic collapse, and autonomic surge intersect. In this high-acuity environment, the cardiac intensivist serves as the vanguard of stability, managing the delicate balance of deep sedation, mechanical ventilation, and the initiation of MCS. The electrophysiologist has the necessary time to perform high-density 3D mapping and substrate modification, which would otherwise be impossible in a crashing patient.

This synergy extends beyond the EP lab. The integration of imaging specialists allows for the pre-procedural fusion of LGE-MRI or CT data, effectively providing a roadmap of the myocardial scar and its heterogeneous border zones. Meanwhile, the cardiac surgeon remains an essential tether for surgical rescue or advanced denervation techniques when percutaneous efforts are exhausted.

**Figure 1:**
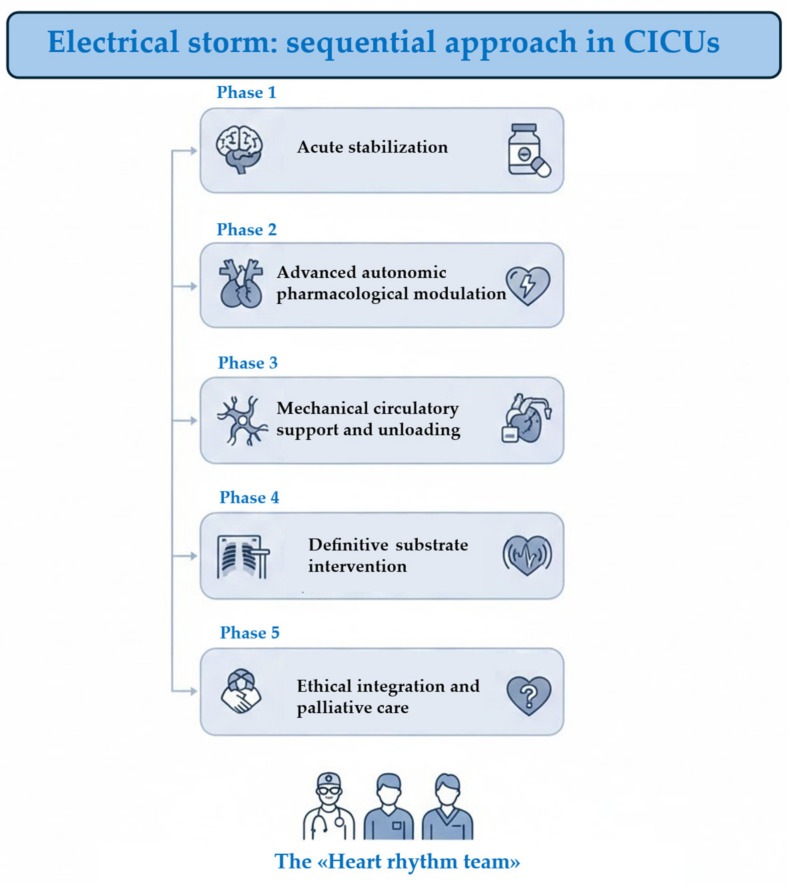
Sequential approach for management of ES in CICUs. Phase 1 focuses on the immediate medical response to stabilize the patient’s condition. Phase 2 involves using specific medications to regulate the nervous system’s influence on the heart. Phase 3 utilizes devices to assist heart function and reduce the workload on the cardiac muscle. Phase 4 targets the underlying physical cause of the arrhythmia, often through procedures like ablation. Phase 5 addresses long-term quality of life, ethical considerations, and supportive care for the patient. CICUs: cardiac intensive care units.

**Figure 2:**
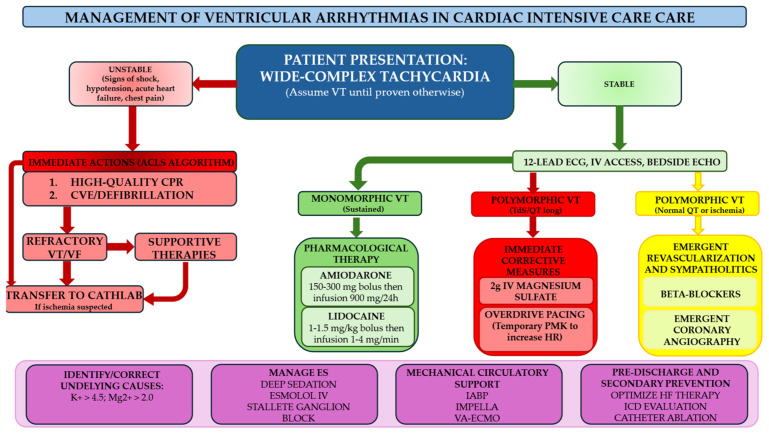
Flowchart of management of VAs in CICUs. ACLS: advanced cardiovascular life support; ECG: electrocardiogram; ES: electrical storm; IABP: intra aortic balloon pump; IV: intravenous; HF: heart failure; HR: heart rate; PMK: pacemaker; TdS: torsade de pointes; VA-ECMO: venoarterial extracorporeal membrane oxygenation; VF: ventricular fibrillation; VT: ventricular arrhythmias.

Crucially, the team approach addresses the ethical and longitudinal complexities of ES. By formalizing this Stepwise Escalation Protocol, the Heart Rhythm Team transforms a chaotic clinical emergency into a structured, precision-driven intervention, ultimately defining the threshold between salvage and survival. Figure 1 represents the proposal approach regarding the heart rhythm team, while Figure 2 represents the flowchart of management of VAs in CICUs.

### 7.2. Palliative Care Integration and End-of-Life Decisions for ICD Patients in Refractory Shock

For patients equipped with an ICD, refractory shock represents a form of medical distress. In the setting of an incessant storm, the device may deliver dozens of painful, high-voltage shocks that no longer offer a survival benefit. Instead, these discharges serve only to prolong the dying process and exacerbate both patient suffering and family trauma. Current guidelines and consensus statements [45] emphasize that deactivating ICD tachyarrhythmia therapies is both ethically and legally distinct from physician-assisted suicide or euthanasia. Rather, it constitutes the withdrawal of a life-sustaining treatment that has become disproportionate, burdensome, and futile in the face of an irreversible clinical decline.

The transition to palliative management requires a highly coordinated narrative, bridging the technical expertise of the electrophysiologist with the critical care focus of the intensivist and the symptom-management mastery of the palliative specialist.

## 8. Limitations

While this review provides a comprehensive synthesis of the evolving strategies for managing VAs in the CICU, several limitations must be acknowledged. First, as a narrative review, this work does not follow the strict methodological protocols of a systematic review or meta-analysis. From a clinical and scientific perspective, several gaps in the current evidence base remain.

Although high-fidelity 3D electroanatomical mapping and image-guided ablation have revolutionized the identification of arrhythmic substrates, their practical application is often limited in high-acuity settings. As well as the application of certain therapeutic techniques, such as STAR, and the role of specific MCS platforms, remains poorly defined and largely anecdotal. Several discussed anti-inflammatory and anti-fibrotic strategies, are still in preclinical stages or early clinical experimentation, requiring further validation before they can be integrated into standard care.

Furthermore, a significant limitation remains the marked heterogeneity in healthcare infrastructure; to overcome this, it is essential to establish dedicated clinical pathways and specialized networks capable of centralizing the management of complex ventricular arrhythmias within high-volume, technologically advanced centers.

## 9. Conclusions

The management of VA within the CICUs has undergone a profound metamorphosis, evolving from a reactive, rhythm-centric approach into a proactive, integrated paradigm. The cornerstone of modern VA management lies in the rapid and systematic stabilization of the electrical and hemodynamic milieu. The success of these advanced interventions is inextricably linked to the heart rhythm team. In conclusion, the modern management of VA in the CICU represents a convergence of technology, biology, and multidisciplinary coordination, collectively defining the threshold between salvage and survival in the contemporary era of cardiac intensive care.

## Figures and Tables

**Table 1:** List of antiarrhythmic drugs for VAs [25].

Acute Treatment										
Vaughan Williams Class	Drug	Channels Affected	Dose (i.v.)	Pharmacokinetics			Specific Use	Caution	Monitoring	Adverse Effects
				Half-Life	Desired Plasma Concentration	Metabolism				
Class I	Quinidine	I_Na_, I_Kr_, I_To_, M, α	Loading dose: 800 mg/50 mL Maintenance intravenous: 50 μg/min	6–8 h	1.5–3.5 μg/mL	Hepatic	VT, VF, ES after MI, PCI, CABG, BrS, SQT syndrome IVF	Severe bradycardia and/or high-degree AV block in the absence of a pacemaker, myasthenia gravis, decompensated heart failure	QRS duration and QT interval, heart rate, platelet count	QTc prolongation, TdP, QRS prolongation, increase in defibrillation threshold, hypotension, bradycardia, heart failure exacerbation, diarrhoea, and immune thrombocytopenia
	Procainamide	I_Na_, I_Kr_ ganglionic block	100 mg bolus, can be repeated after 5 min if no effect, alternatively 10–17 mg/kg administered at a rate of 20–50 mg/min, max 500–750 mg (max 50 mg/min) and then 2–6 mg/min	3–4 h	4–12 μg/mL	Hepatic and renal	VT, pre-excited AF	Severe sinus node disease, severe AV conduction disturbance, previous MI, reduced LVEF, hypotension, BrS, myasthenia gravis	QRS duration and QT interval, blood pressure	Rash, myalgia, vasculitis, agranulocytosis, hypotension, bradycardia, QT prolongation
	Lidocaine	I_Na_	50–200 mg bolus and then 2–4 mg/min (25–50 μg/kg/min)	7–30 min	2–6 μg/mL	Hepatic	Ischaemic VT/VF	Severe sinus node/AV heart block in the absence of pacemaker, cardiogenic shock, hypersensitivity to lidocaine or amide-type local anaesthetist	PR interval and QRS duration, temperature	Hypotension, confusion, tremors, methemoglobinaemia, malignant hyperthermia, anaphylactoid reactions
	Mexiletine	I_Na_	Intravenous: not recommended Loading dose: 400 mg initially followed by 600 mg in the first 24 h Maintenance dose: 600–1200 mg	10–14 h	0.6–1.7 mg/mL	Hepatic	VT/VF, LQT3 LQT2	Cardiogenic shock, pre-existing sinus node disease, or second/third-degree AV block without pacemaker, hypotension, history of seizures	QT interval, hepatic function	Ataxia, tremors, hypotension, angina
Class II (BBs)	Esmolol	β1-receptor	Bolus dose: 0.5 mg/kg for 1 min Infusion: 25–50 μg/kg/min up to 250 μg/kg/min (titrate every 5–10 min)	5–10 min	NA	RBC esterase	VT	Severe sinus bradycardia/severe sinus node disease/AV conduction disturbances without pacemaker, Decompensated heart failure, Prinzmetal’s angina, asthma/chronic obstructive airway disease, myasthenia gravis	Heart rate, blood pressure	Bronchospasm, hypotension, sinus bradycardia, AV block, fatigue, depression
	Propranolol	Non-selective BB	Bolus dose: 0.15 mg/kg over 10 min, 160 mg/24 h	3–6 h	NA	Hepatic	VT, PVCs, LQT			
	Metoprolol	β1-receptor	Bolus dose: 2–5 mg every 5 min, up to 3 doses in 15 min	Tartarate: 3–4 h Succinate: 3–7 h	NA	Hepatic	VT, PVCs			
Class III	Amiodarone	I_Na_, I_Ca,_ I_Kr_, I_K1_, I_Ks_, I_to_, α, β	Loading dose: 5 mg/kg in 20 min to 2 h, 2–3 times in 24 h and then 600–1200 mg/24 h 8–10 days	4–14 weeks	1–2.5 μg/mL	Hepatic	VT, VF, PVCs	Concomitant digoxin or warfarin administration	Heart rate, blood pressure	Increases DFT, hypotension, bradycardia, AV block, QT prolongation, TdP (rare), hypothyroidism/hyperthyroidism, nausea, photosensitivity, skin discolouration, peripheral neuropathy, tremor, hepatitis, pulmonary fibrosis/pneumonitis
Beta agonist	Isoproterenol	β1 and β2	0.5–10 μg/min	2.5–5 min	NA	Hepatic and pulmonary	IVF TdP, ES in Brugada syndrome, SQT syndrome, VAs secondary to AV block	Coronary artery disease, MI, convulsions, renal disease, hyperthyroidism	Heart rate, blood pressure, ST-elevation	Tachycardia, hypertension, angina, tremors

AF: atrial fibrillation; BB; beta-blocker; BrS; Brugada syndrome; CABG: coronary artery bypass grafting; DFT: defibrillation threshold testing; ECG: electrocardiogram; ES: electrical storm; IVF: idiopathic ventricular fibrillation; LVEF: left ventricular ejection fraction; LQT: long QT; MI: myocardial infarction; NA: not applicable; PCI: percutaneous coronary intervention; PVC, premature ventricular complex; QTc: corrected QT; RBC: red blood cell; SQT: short QT; TdP: torsade de pointes; VF: ventricular fibrillation; VAs: ventricular arrhythmias; VT: ventricular tachycardia.

**Table 2:** Main drug for sedation regarding the management of VAs [25].

Agents	Initial Dose	Infusion Rate
Benzodiazepines		
Midazolam	0.01–0.05 mg/kg, repeat q5–15 min	0.02–0.1 mg/kg/h, titrate up/down by 25–50%
Lorazepam	0.02–0.04 mg/kg	0.01–0.1 mg/kg/h (not exceed 10 mg/h)
Propofol	0.8–1.2 mg/kg	5–50 μg/kg/min (0.3–3 mg/kg/h)
		Up-titrate every 5–10 min by 5–10 μg/kg/h
Opioids		
Fentanyl	1–2 μg/kg	1–2 μg/kg/h
Remifentanyl	0.5–1.5 μg/kg	0.05–2 μg/kg/min
Dexmedetomidine	1 μg/kg over 10 min	0.2–0.7 μg/kg/h

**Table 3:** Main characteristics of MCS [33,34].

Type of MCS	Mechanism of MCS	Technical Advantages	Clinical Advantages	Clinical Disvantages	Contraindications
IABP	Indirect LV support by aortic counterpulsation, with afterload reduction and diastolic aortic pressure augmentation	Percutaneous arterial access (7–8 Fr) Modest cardiac output increase (0.5–1.0 L/min)	Simple set-up Indirectly unloads LV Availability Affordable Low complication rate	Very limited LV support Requires stable rhythm	Moderate-severe aortic regurgitation Severe peripheral artery disease Aortic dissection
Impella CP	Microaxial continuous anterograde flow pump LV aorta	Maximum average flow 3.7 L/min Percutaneous arterial access (femoral/axillary) Introducer diameter 14 Fr Pump motor 14 Fr	Relatively simple set-up and implantation Unloads LV Availability Percutaneous removal	Partial LV support Electromagnetic interference in mapping system. Difficult catheter manipulation during ablation May require transeptal access for CA High costs	Moderate-severe aortic regurgitation Mechanic aortic prosthesis LV thrombus Severe peripheral artery disease Severe RV failure
Impella 5.5	Microaxial continuous anterograde flow pump LV aorta	Maximum average flow 5.5 L/min Surgical axillary cut-down Introducer diameter 23 Fr Pump motor 19 Fr	Full LV support Unloads LV Prolonged support duration Allows early mobilization	Requires surgical team/complex logistics Surgical insertion and removal High costs	Moderate-severe aortic regurgitation Mechanic aortic prosthesis LV thrombus Severe peripheral artery disease Severe RV failure
Impella RP	Type of MCS	Main characteristics	Technical details	Anecdotical experience in ES or VT ablation	Mechanical valves, severe valvular stenosis/regurgitation of the tricuspid or pulmonary valve
				Only available in selected sites High costs	Right atrium or vena cava thrombus Presence of a vena cava filter or caval interruption device
TandemHeart	Left atrial-to-femoral artery bypass using external centrifugal pump	Percutaneous or surgical access Transseptal puncture required Generated output up to 5 L/min Inflow cannula 21 Fr Outflow cannula 15 Fr	LV support Indirectly unloads LV Prolonged support duration	Complex logistics Multi-disciplinary team May impede transeptal approach for CA Large bore cannulas	Moderate-severe aortic regurgitation RV failure Ventricular septal defect Severe peripheral artery disease
VA-ECMO	Cardiopulmonary support system integrated by a centrifugal pump and an oxygenation membrane	Percutaneous/surgical vascular access 17–21 Fr outflow cannula 19–25 Fr inflow cannula Maximum output up to 8 L/min	Full biventricular support Prolonged support duration Default choice in isolated RV failure May be percutaneously removed	Complex logistics Multi-disciplinary team Requires often arterial distal perfusion cannula to prevent leg ischaemia High complication rates	Severe aortic regurgitation Aortic dissection Severe peripheral artery disease (in peripheral cannulation) Uncontrollable bleeding or contraindications to systemic anticoagulation

CA: catheter ablation; ES: electrical storm; IABP: intra-aortic balloon pump; LV: left ventricle; MCS: mechanical circulatory support; RV: right ventricle; VA-ECMO: veno-arterial extracorporeal membrane oxygenation; VT: ventricular tachycardia.

**Table 4:** PAINESD Score.

Parameter	Points
Pulmonary disease (COPD)	5
Age > 60 years	3
IHD	6
NYHA Class III and IV	6
EF < 25%	3
Storm electrical	5
DM	3
Low risk: ≤8, median risk: 9–14, high risk: ≥15 points	

COPD: chronic obstructive pulmonary disease; DM: diabetes mellitus; EF: ejection fraction; IHD: ischaemic heart disease; NYHA: New York Heart Association.

## Data Availability

No new data were created or analyzed in this study.

## Abbreviations

BB	Beta-Blocker
CICU	Cardiac Intensive Care Unit
ES	Electrical Storm
IABP	Intra-Aortic Balloon Pump
ICD	Implantable Cardioverter Defibrillator
MCS	Mechanical Circulatory Support
VAs	Ventricular Arrhythmias
VA-ECMO	Veno-Arterial Extracorporeal Membrane Oxygenation
VF	Ventricular Fibrillation
VT	Ventricular Tachycardia

## References

[1] Fuster V. (2018). The (R) Evolution of the CICU: Better for the patient, better for education. J. Am. Coll. Cardiol..

[2] Sinha S.S., Sjoding M.W., Sukul D., Prescott H.C., Iwashyna T.J., Gurm H.S., Cooke C.R., Nallamothu B.K. (2017). Changes in Primary Noncardiac Diagnoses Over Time Among Elderly Cardiac Intensive Care Unit Patients in the United States. Circ. Cardiovasc. Qual. Outcomes.

[3] Watson R.A., Bohula E.A., Gilliland T.C., Sanchez P.A., Berg D.D., Morrow D.A. (2019). Editor’s Choice-Prospective Registry of Cardiac Critical Illness in a Modern Tertiary Care Cardiac Intensive Care Unit. Eur. Heart J. Acute Cardiovasc. Care.

[4] Artucio H., Pereira M. (1990). Cardiac Arrhythmias in Critically Ill Patients. Crit. Care Med..

[5] Reinelt P., Karth G., Geppert A., Heinz G. (2001). Incidence and Type of Cardiac Arrhythmias in Critically Ill Patients: A Single Center Experience in a Medical-Cardiological ICU. Intensive Care Med..

[6] Valderrábano R.J., Blanco A., Santiago-Rodriguez E.J., Miranda C., Rivera-del Rio del Rio J., Ruiz J., Hunter R. (2016). Risk Factors and Clinical Outcomes of Arrhythmias in the Medical Intensive Care Unit. J. Intensive Care.

[7] Elsokkari I., Parkash R., Tang A., Wells G., Doucette S., Yetisir E., Gardner M., Healey J.S., Thibault B., Sterns L. (2020). Mortality Risk Increases With Clustered Ventricular Arrhythmias in Patients With Implantable Cardioverter-Defibrillators. JACC Clin. Electrophysiol..

[8] Mauriello A., Correra A., Del Vecchio G.E., Grieco M., Amata A., Di Micco P., Imbalzano E., Paternoster M., Ascrizzi A., Quagliariello V. (2025). Heart Failure and Wide QRS: Clinical and Pharmacological Perspectives. Biomedicines.

[9] Nademanee K., Taylor R., Bailey W.E., Rieders D.E., Kosar E.M. (2000). Treating Electrical Storm: Sympathetic blockade versus advanced cardiac life support–guided therapy. Circulation.

[10] Chatzidou S., Kontogiannis C., Tsilimigras D.I., Georgiopoulos G., Kosmopoulos M., Papadopoulou E., Vasilopoulos G., Rokas S. (2018). Propranolol Versus Metoprolol for Treatment of Electrical Storm in Patients With Implantable Cardioverter-Defibrillator. J. Am. Coll. Cardiol..

[11] Miwa Y., Ikeda T., Mera H., Miyakoshi M., Hoshida K., Yanagisawa R., Ishiguro H., Tsukada T., Abe A., Yusu S. (2010). Effects of Landiolol, an Ultra-Short-Acting β_1_-Selective Blocker, on Electrical Storm Refractory to Class III Antiarrhythmic Drugs. Circ. J..

[12] Ikeda T., Shiga T., Shimizu W., Kinugawa K., Sakamoto A., Nagai R., Daimon T., Oki K., Okamoto H., Yamashita T. (2019). Efficacy and Safety of the Ultra-Short-Acting β1-Selective Blocker Landiolol in Patients With Recurrent Hemodynamically Unstable Ventricular Tachyarrhymias—Outcomes of J-Land II Study. Circ. J..

[13] Shiga T., Shiozaki M., Takahashi R., Matsumoto R., Fukui M. (2025). Post-Marketing Surveillance of the Safety of Landiolol in Patients With Recurrent Hemodynamically Unstable Ventricular Tachyarrhythmias. Circ. J..

[14] Ortiz M., Martín A., Arribas F., Coll-Vinent B., del Arco C., Peinado R., Almendral J. (2016). Randomized Comparison of Intravenous Procainamide vs. Intravenous Amiodarone for the Acute Treatment of Tolerated Wide QRS Tachycardia: The PROCAMIO Study. Eur. Heart J..

[15] Kerin N.Z., Blevins R.D., Frumin H., Faitel K., Rubenfire M. (1985). Intravenous and Oral Loading versus Oral Loading Alone with Amiodarone for Chronic Refractory Ventricular Arrhythmias. Am. J. Cardiol..

[16] Muser D., Santangeli P., Liang J.J. (2017). Management of Ventricular Tachycardia Storm in Patients with Structural Heart Disease. World J. Cardiol..

[17] Gorgels A.P.M., van den Dool A., Hofs A., Mulleneers R., Smeets J.L.R.M., Vos M.A., Wellens H.J.J. (1996). Comparison of Procainamide and Lidocaine in Terminating Sustained Monomorphic Ventricular Tachycardia. Am. J. Cardiol..

[18] Ho D. (1994). Double-Blind Trial of Lignocaine versus Sotalol for Acute Termination of Spontaneous Sustained Ventricular Tachycardia. Lancet.

[19] Tsuboi M., Chiba S. (1999). Effects of Lidocaine on Isolated, Blood-Perfused Ventricular Contractility in the Dog. Heart Vessel..

[20] Antzelevitch C., Burashnikov A., Sicouri S., Belardinelli L. (2011). Electrophysiologic Basis for the Antiarrhythmic Actions of Ranolazine. Heart Rhythm.

[21] Tzivoni D., Banai S., Schuger C., Benhorin J., Keren A., Gottlieb S., Stern S. (1988). Treatment of Torsade de Pointes with Magnesium Sulfate. Circulation.

[22] Ohgo T., Okamura H., Noda T., Satomi K., Suyama K., Kurita T., Aihara N., Kamakura S., Ohe T., Shimizu W. (2007). Acute and Chronic Management in Patients with Brugada Syndrome Associated with Electrical Storm of Ventricular Fibrillation. Heart Rhythm.

[23] Bun S., Maury P., Giustetto C., Deharo J. (2012). Electrical Storm in Short-QT Syndrome Successfully Treated with Isoproterenol. J. Cardiovasc. Electrophysiol..

[24] Bellamy D., Nuthall G., Dalziel S., Skinner J.R. (2019). Catecholaminergic Polymorphic Ventricular Tachycardia: The Cardiac Arrest Where Epinephrine Is Contraindicated*. Pediatr. Crit. Care Med..

[25] Könemann H., Ellermann C., Zeppenfeld K., Eckardt L. (2023). Management of Ventricular Arrhythmias Worldwide. JACC Clin. Electrophysiol..

[26] Ajijola O.A., Aksu T., Arora R., Biaggioni I., Chen P., De Ferrari G., Dusi V., Fudim M., Goldberger J.J., Green A.L. (2025). Clinical Neurocardiology: Defining the Value of Neuroscience-based Cardiovascular Therapeutics–2024 Update. J. Physiol..

[27] Martins R.P., Urien J.-M., Barbarot N., Rieul G., Sellal J.-M., Borella L., Clementy N., Bisson A., Guenancia C., Sagnard A. (2020). Effectiveness of Deep Sedation for Patients With Intractable Electrical Storm Refractory to Antiarrhythmic Drugs. Circulation.

[28] Bundgaard J.S., Jacobsen P.K., Grand J., Lindholm M.G., Hassager C., Pehrson S., Kjaergaard J., Bundgaard H. (2020). Deep Sedation as Temporary Bridge to Definitive Treatment of Ventricular Arrhythmia Storm. Eur. Heart J. Acute Cardiovasc. Care.

[29] Vaseghi M., Gima J., Kanaan C., Ajijola O.A., Marmureanu A., Mahajan A., Shivkumar K. (2014). Cardiac Sympathetic Denervation in Patients with Refractory Ventricular Arrhythmias or Electrical Storm: Intermediate and Long-Term Follow-Up. Heart Rhythm.

[30] Savastano S., Baldi E., Compagnoni S., Rordorf R., Sanzo A., Gentile F.R., Dusi V., Frea S., Gravinese C., Cauti F.M. (2024). Electrical Storm Treatment by Percutaneous Stellate Ganglion Block: The STAR Study. Eur. Heart J..

[31] Bazoukis G., Korantzopoulos P., Tsioufis C. (2016). The Impact of Renal Sympathetic Denervation on Cardiac Electrophysiology and Arrhythmias: A Systematic Review of the Literature. Int. J. Cardiol..

[32] Do D.H., Bradfield J., Ajijola O.A., Vaseghi M., Le J., Rahman S., Mahajan A., Nogami A., Boyle N.G., Shivkumar K. (2017). Thoracic Epidural Anesthesia Can Be Effective for the Short-Term Management of Ventricular Tachycardia Storm. J. Am. Heart Assoc..

[33] Atti V., Narayanan M.A., Patel B., Balla S., Siddique A., Lundgren S., Velagapudi P. (2022). A Comprehensive Review of Mechanical Circulatory Support Devices. Heart Int..

[34] Tavazzi G., Dammassa V., Colombo C.N.J., Arbustini E., Castelein T., Balik M., Vandenbriele C. (2022). Mechanical Circulatory Support in Ventricular Arrhythmias. Front. Cardiovasc. Med..

[35] Mauriello A., Marrazzo G., Del Vecchio G.E., Ascrizzi A., Roma A.S., Correra A., Sabatella F., Gioia R., Desiderio A., Russo V. (2024). Echocardiography in Cardiac Arrest: Incremental Diagnostic and Prognostic Role during Resuscitation Care. Diagnostics.

[36] Yannopoulos D., Bartos J., Raveendran G., Walser E., Connett J., Murray T.A., Collins G., Zhang L., Kalra R., Kosmopoulos M. (2020). Advanced Reperfusion Strategies for Patients with Out-of-Hospital Cardiac Arrest and Refractory Ventricular Fibrillation (ARREST): A Phase 2, Single Centre, Open-Label, Randomised Controlled Trial. Lancet.

[37] Belohlavek J., Smalcova J., Rob D., Franek O., Smid O., Pokorna M., Horák J., Mrazek V., Kovarnik T., Zemanek D. (2022). Effect of Intra-Arrest Transport, Extracorporeal Cardiopulmonary Resuscitation, and Immediate Invasive Assessment and Treatment on Functional Neurologic Outcome in Refractory Out-of-Hospital Cardiac Arrest. JAMA.

[38] Suverein M.M., Delnoij T.S.R., Lorusso R., Brandon Bravo Bruinsma G.J., Otterspoor L., Elzo Kraemer C.V., Vlaar A.P.J., van der Heijden J.J., Scholten E., den Uil C. (2023). Early Extracorporeal CPR for Refractory Out-of-Hospital Cardiac Arrest. N. Engl. J. Med..

[39] Kalra R., Yannopoulos D., Bartos J.A. (2024). Left Ventricular Unloading during VA-ECMO: A Gordian Knot of Physiology. Resuscitation.

[40] Enriquez A., Liang J., Gentile J., Schaller R.D., Supple G.E., Frankel D.S., Garcia F.C., Wald J., Birati E.Y., Rame J.E. (2018). Outcomes of Rescue Cardiopulmonary Support for Periprocedural Acute Hemodynamic Decompensation in Patients Undergoing Catheter Ablation of Electrical Storm. Heart Rhythm.

[41] Chung F., Liao Y., Lin Y., Chang S., Lo L., Hu Y., Tuan T., Chao T., Liao J., Lin C. (2020). Outcome of Rescue Ablation in Patients with Refractory Ventricular Electrical Storm Requiring Mechanical Circulation Support. J. Cardiovasc. Electrophysiol..

[42] Mariani S., Napp L.C., Lo Coco V., Delnoij T.S.R., Luermans J.G.L.M., ter Bekke R.M.A., Timmermans C., Li T., Dogan G., Schmitto J.D. (2020). Mechanical Circulatory Support for Life-Threatening Arrhythmia: A Systematic Review. Int. J. Cardiol..

[43] Muser D., Liang J.J., Castro S.A., Hayashi T., Enriquez A., Troutman G.S., McNaughton N.W., Supple G., Birati E.Y., Schaller R. (2018). Outcomes with Prophylactic Use of Percutaneous Left Ventricular Assist Devices in High-Risk Patients Undergoing Catheter Ablation of Scar-Related Ventricular Tachycardia: A Propensity-Score Matched Analysis. Heart Rhythm.

[44] Luni F.K., Zungsontiporn N., Farid T., Malik S.A., Khan S., Daniels J., Wu R., Link M.S., Joglar J.A. (2019). Percutaneous Left Ventricular Assist Device Support during Ablation of Ventricular Tachycardia: A Meta-analysis of Current Evidence. J. Cardiovasc. Electrophysiol..

[45] Zeppenfeld K., Tfelt-Hansen J., de Riva M., Winkel B.G., Behr E.R., Blom N.A., Charron P., Corrado D., Dagres N., de Chillou C. (2022). 2022 ESC Guidelines for the Management of Patients with Ventricular Arrhythmias and the Prevention of Sudden Cardiac Death. Eur. Heart J..

[46] Bhaskaran A., Tung R., Stevenson W.G., Kumar S. (2019). Catheter Ablation of VT in Non-Ischaemic Cardiomyopathies: Endocardial, Epicardial and Intramural Approaches. Heart Lung Circ..

[47] Priori S.G., Wilde A.A., Horie M., Cho Y., Behr E.R., Berul C., Blom N., Brugada J., Chiang C.-E., Huikuri H. (2013). HRS/EHRA/APHRS Expert Consensus Statement on the Diagnosis and Management of Patients with Inherited Primary Arrhythmia Syndromes. Heart Rhythm.

[48] Dragasis S., Vlachos K., Frontera A., Mililis P., Saplaouras A., Zygouri A., Zymatoura M.E., Kontonika M., Kafkas N., Efremidis M. (2022). Modern Mapping and Ablation of Idiopathic Outflow Tract Ventricular Arrhythmias. Rev. Cardiovasc. Med..

[49] Compagnucci P., Volpato G., Falanga U., Cipolletta L., Conti M., Grifoni G., Verticelli L., Schicchi N., Giovagnoni A., Casella M. (2021). Recent Advances in Three-Dimensional Electroanatomical Mapping Guidance for the Ablation of Complex Atrial and Ventricular Arrhythmias. J. Interv. Card. Electrophysiol..

[50] Thibault B., Richer L.-P., McSpadden L.C., Ryu K., Aguilar M., Cadrin-Tourigny J., Tadros R., Mondésert B., Rivard L., Dyrda K. (2022). Integration of 3D Nuclear Imaging in 3D Mapping System for Ventricular Tachycardia Ablation in Patients with Implanted Devices: Perfusion/Voltage Retrospective Assessment of Scar Location. Heart Rhythm O^2^.

[51] Mauriello A., Correra A., Ascrizzi A., Del Vecchio G.E., Benfari G., Ilardi F., Lisi M., Malagoli A., Mandoli G.E., Pastore M.C. (2025). Relationship Between Left Atrial Strain and Atrial Fibrillation: The Role of Stress Echocardiography. Diagnostics.

[52] Mukherjee R.K., Whitaker J., Williams S.E., Razavi R., O’Neill M.D. (2018). Magnetic Resonance Imaging Guidance for the Optimization of Ventricular Tachycardia Ablation. EP Eur..

[53] Stevens S.M., Tung R., Rashid S., Gima J., Cote S., Pavez G., Khan S., Ennis D.B., Finn J.P., Boyle N. (2014). Device Artifact Reduction for Magnetic Resonance Imaging of Patients with Implantable Cardioverter-Defibrillators and Ventricular Tachycardia: Late Gadolinium Enhancement Correlation with Electroanatomic Mapping. Heart Rhythm.

[54] Andreu D., Penela D., Acosta J., Fernández-Armenta J., Perea R.J., Soto-Iglesias D., de Caralt T.M., Ortiz-Perez J.T., Prat-González S., Borràs R. (2017). Cardiac Magnetic Resonance–Aided Scar Dechanneling: Influence on Acute and Long-Term Outcomes. Heart Rhythm.

[55] van der Ree M.H., Dieleman E.M.T., Visser J., Planken R.N., Boekholdt S.M., de Bruin-Bon R.H.A., Rasch C.R.N., Hoeksema W.F., de Jong R.M.A.J., Kemme M.J.B. (2023). Non-Invasive Stereotactic Arrhythmia Radiotherapy for Ventricular Tachycardia: Results of the Prospective STARNL-1 Trial. EP Eur..

[56] Herrera Siklody C., Schiappacasse L., Jumeau R., Reichlin T., Saguner A.M., Andratschke N., Elicin O., Schreiner F., Kovacs B., Mayinger M. (2023). Recurrences of Ventricular Tachycardia after Stereotactic Arrhythmia Radioablation Arise Outside the Treated Volume: Analysis of the Swiss Cohort. Europace.

[57] Mauriello A., Ascrizzi A., Molinari R., Falco L., Caturano A., D’Andrea A., Russo V. (2023). Pharmacogenomics of Cardiovascular Drugs for Atherothrombotic, Thromboembolic and Atherosclerotic Risk. Genes.

[58] Roden D.M. (2014). Personalized Medicine to Treat Arrhythmias. Curr. Opin. Pharmacol..

[59] Li S., Lv T., Yang J., Li K., Yang Y., Zhang P. (2023). A Gain of Function Ryanodine Receptor 2 Mutation (R1760W-RyR2) in Catecholaminergic Polymorphic Ventricular Tachycardia. Clin. Exp. Pharmacol. Physiol..

[60] Biakina O., Mitina Y., Gognieva D., Axenova M., Ermolaeva A., Bestavashvili A., Fadeev V., Syrkin A., Kopylov P. (2023). DUOX1 Gene Missense Mutation Confers Susceptibility on Type 2 Amiodarone-Induced Thyrotoxicosis. Int. J. Mol. Sci..

[61] Baksi A.J., Kanaganayagam G.S., Prasad S.K. (2015). Arrhythmias in Viral Myocarditis and Pericarditis. Card. Electrophysiol. Clin..

[62] Park H., Park H., Lee D., Oh S., Lim J., Hwang H.j., Park S., Pak H.-N., Lee M.-H., Joung B. (2014). Increased Phosphorylation of Ca^2+^ Handling Proteins as a Proarrhythmic Mechanism in Myocarditis. Circ. J..

[63] He Y., Hara H., Núñez G. (2016). Mechanism and Regulation of NLRP3 Inflammasome Activation. Trends Biochem. Sci..

[64] Ho H., Stevens S.C.W., Terentyeva R., Carnes C.A., Terentyev D., Györke S. (2011). Arrhythmogenic Adverse Effects of Cardiac Glycosides Are Mediated by Redox Modification of Ryanodine Receptors. J. Physiol..

[65] Cooper L.L., Li W., Lu Y., Centracchio J., Terentyeva R., Koren G., Terentyev D. (2013). Redox Modification of Ryanodine Receptors by Mitochondria-derived Reactive Oxygen Species Contributes to Aberrant Ca^2+^ Handling in Ageing Rabbit Hearts. J. Physiol..

[66] Belevych A.E., Terentyev D., Viatchenko-Karpinski S., Terentyeva R., Sridhar A., Nishijima Y., Wilson L.D., Cardounel A.J., Laurita K.R., Carnes C.A. (2009). Redox Modification of Ryanodine Receptors Underlies Calcium Alternans in a Canine Model of Sudden Cardiac Death. Cardiovasc. Res..

[67] Terentyev D., Györke I., Belevych A.E., Terentyeva R., Sridhar A., Nishijima Y., Carcache de Blanco E., Khanna S., Sen C.K., Cardounel A.J. (2008). Redox Modification of Ryanodine Receptors Contributes to Sarcoplasmic Reticulum Ca ^2+^ Leak in Chronic Heart Failure. Circ. Res..

[68] Donahue J.K., Chrispin J., Ajijola O.A. (2024). Mechanism of Ventricular Tachycardia Occurring in Chronic Myocardial Infarction Scar. Circ. Res..

[69] Bahia M.S., Kaur M., Silakari P., Silakari O. (2015). Interleukin-1 Receptor Associated Kinase Inhibitors: Potential Therapeutic Agents for Inflammatory- and Immune-Related Disorders. Cell. Signal..

[70] Lv Z., Chen X., Chen P., Li Q., Guo Z., Lu Q., Ding S. (2022). Colchicine Prevents Ventricular Arrhythmias Vulnerability in Diet-Induced Obesity Rats. Biochem. Biophys. Res. Commun..

[71] Nguyen D.T., Ding C., Wilson E., Marcus G.M., Olgin J.E. (2010). Pirfenidone Mitigates Left Ventricular Fibrosis and Dysfunction after Myocardial Infarction and Reduces Arrhythmias. Heart Rhythm.

